# A Chiral-LC-MS Method for the Simultaneous Quantification of Short-Chain Fatty Acids and D/L-Lactate in the Ruminal Fluid of Dairy Cows

**DOI:** 10.3390/molecules29061398

**Published:** 2024-03-21

**Authors:** Zhiqian Liu, S. Richard O. Williams, Joe L. Jacobs, Aodan S. O. Neachtain, Simone Rochfort

**Affiliations:** 1 Agriculture Victoria Research, AgriBio, 5 Ring Road, Bundoora, VIC 3083, Australia; simone.rochfort@agriculture.vic.gov.au; 2 Agriculture Victoria Research, Ellinbank SmartFarm, Ellinbank, VIC 3821, Australia; richard.williams@agriculture.vic.gov.au (S.R.O.W.); joe.jacobs@agriculture.vic.gov.au (J.L.J.); aodan.o.neachtain@agriculture.vic.gov.au (A.S.O.N.); 3 School of Agriculture, Food and Ecosystem Sciences, Faculty of Science, The University of Melbourne, Melbourne, VIC 3010, Australia; 4 School of Applied Systems Biology, La Trobe University, Bundoora, VIC 3083, Australia

**Keywords:** short-chain fatty acids, D-lactate, L-lactate, ruminal fluid, liquid chromatography–mass spectrometry

## Abstract

Short-chain fatty acids (SCFA) and lactate in ruminal fluid are products resulting from the microbial fermentation of substrates and can be used to reflect the composition and activity of the ruminal microbiome. Determination of SCFA and D-/L-lactate in ruminal fluid currently requires two separate protocols, which is time-consuming and costly. In this study, we have optimised and validated a simple and unified 3-nitrophenylhydrazine (3-NPH) derivatisation protocol and a 20 min chiral-LC-MS method for the simultaneous quantification of all SCFA and D- and L-lactate in ruminal fluid. This method, which requires no sample pretreatment or purification shows adequate sensitivity (limit of detection (LOD): 0.01 µg/mL), satisfactory accuracy (recovery: 88–103%), and excellent reproducibility (relative standard deviation (RSD) for repeated analyses < 3% for most analytes). The application of this method to a cohort of 24 animals allowed us to reveal a large inter-cow variation in ruminal SCFA and lactate level, the concentration range for each species, the widespread correlation between different SCFA, and the strong correlation between D- and L-lactate.

## 1. Introduction

Short-chain fatty acids (SCFA), also called volatile fatty acids (VFA) are the products of feed fermentation in the rumen [1], whereas D-/L-lactate are the intermediate products of starch fermentation in the rumen [2]. Given ruminal SCFA and lactate profiles are associated with ruminant feeding regime, ruminal microbial composition, as well as ruminal acidosis severity [1]. The quantification of SCFA and lactate in ruminal liquid is of great importance for monitoring the function of rumen epithelium, energy supply, animal production potential, and animal health.

Numerous methods have been reported over the past two decades for the quantification of SCFA in various matrices, and a number of recent reviews on this topic are available [3,4]. Although nuclear magnetic resonance and capillary electrophoresis have been implemented in SCFA analysis [3], GC and HPLC are by far the most widely used techniques.

For GC-based methods (including GC-FID and GC-MS), two types of protocols can be found for SCFA quantification, i.e., direct GC analysis and GC analysis after derivatisation [5,6,7,8,9,10,11,12]. While a sample pretreatment step (such as acidification, liquid–liquid extraction and solid-phase microextraction) is generally performed for direct GC injection, several derivatisation protocols (such as silylation, methylation and chloroformate derivatisation) have been reported for SCFA. The advantages and disadvantages of various sample pretreatment and derivatisation techniques for the analysis of SCFA by GC have been thoroughly reviewed [3]. Overall, GC methods are robust and popular for SCFA quantification, but sample preparation can be time-consuming and prone to the loss of analytes via evaporation. In addition, specific GC columns are required for the direct injection of underivatized samples. 

LC-MS is increasingly being used in SCFA analysis owing to its faster sample preparation, greater sensitivity, as well as easier access to instruments [4]. Due to the inherently low MS response and poor peak shape of underivatized SCFA molecules, a derivatisation step is usually implemented before RP-LC-MS analysis [13,14,15,16]. Several derivatisation reagents, such as 3-nitrophenylhydrazine (3-NPH), aniline, 2-picolylamine (PA), O-benzylhydroxylamine (O-BHA) and 4-acetoamido-7-mercapto-2,1,3-benzoxadiazole (AABD-SH), have been reported in the literature [4], with the most widely adopted reagent being 3-NPH. Our previous study found that 3-NPH derivatisation is a simple procedure and affords a similar detection sensitivity for branched-chain and linear SCFA molecules present in bovine milk and serum [14].

In the case of D- and L-lactate measurement, in addition to reversed-phase (RP)-LC-MS (after derivatisation) and direct chiral-LC-MS techniques [17,18,19], stereospecific enzymatic methods are widely used, despite the much lower sensitivity and possible cross-enzymatic reactions of the latter [20].

Currently, options are available for separate quantification of SCFA and D/L-lactate in different matrices, but a single method that could measure all these compounds in biological samples would be advantageous. The aim of this study was to establish a robust yet simple method for the simultaneous determination of all linear and branched SCFA and lactate enantiomers in ruminal fluid samples. 

## 2. Results

**Figure 1:**
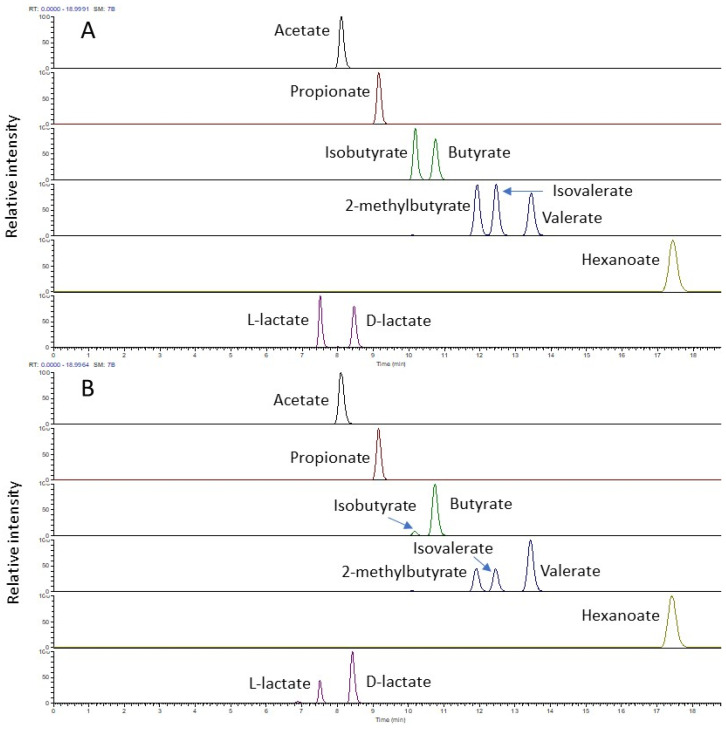
LC-MS profile (extracted ion chromatogram) of 3-NPH derivatised short-chain fatty acids and D/L-lactate in the negative mode from a standard mix (5 µg/mL each) (**A**) and from a ruminal fluid sample (**B**).

The chiral column not only provides excellent separation for the chiral isomer pair D/L-lactate and SCFA with a different carbon number, but also allows all butyrate and valerate isomers present in the standard mix to be baseline resolved within a total runtime of 20 min (Figure 1A). In the ruminal fluid, in addition to D- and L-lactate, acetate, propionate, two isomers for butyrate (C4), three isomers for valerate (C5), and a single linear structure for hexanoate (C6) were found (Figure 1B). For both standards and ruminal fluid samples, a satisfactory peak shape was achieved for all the analytes using this stationary phase.

This chiral-LC-MS method shows good sensitivity for both SCFA and lactate after derivatisation, with the LOD and LOQ being 0.01 and 0.033 µg/mL, respectively for all compounds; the linear range (R^2^ > 0.99) was between 0.033 and 20 µg/mL (Table 1). The calibration curves as well as the regression formulas for all the analytes are shown in Figure S1 (Supporting Information). 

The method shows good intra-day precision (or repeatability), as evidenced by the repeated analysis results of four random ruminal fluid samples on the same day. Although a large variation in SCFA and D/L-lactate concentration was observed across the four samples, the RSD values between repeated measurements were below 3% for most analytes (Table 2). The slightly greater RSD values observed with L-lactate in some samples may be due to the low concentration of this analyte in ruminal fluid.

In addition, the method afforded a satisfactory inter-day precision (or reproducibility). The concentrations of SCFA and D/L-lactate of four random samples determined on two different dates (10 days apart) were very close (difference < 10% for most measurements) (Table S1, Supporting Information).

The presence of the sample matrix did not interfere significantly with the quantification of SCFA and D/L-lactate, as judged by the spike recovery test. When spiked to the 40-fold diluted ruminal fluid, both compounds showed a recovery close to 100% (96.7–102.7%), irrespective of the spike level. A lower recovery (88.2–94.5%) was recorded when these compounds were mixed with a more concentrated sample (20-fold diluted, Table 3).

The validated method was then applied to the ruminal fluid samples of 24 dairy cows. Table 4 shows that SCFA are present at a much greater concentration than D/L-lactate in ruminal fluids, and acetate, propionate, and butyrate are the most abundant SCFA species. Branched-chain isomers (isobutyrate, 2-methylbutyrate, and isovalerate) are less abundant than their straight-chain isomers. Although found at a much lower level, D- and L-lactate were ubiquitously detected in these samples and their concentration were of a similar magnitude (Table 4). In addition, a large inter-cow fluctuation was observed for all SCFA and lactate species, with D- and L-lactate concentration displaying a much greater variation compared to any of the SCFA species, as judged by the RSD values (Table 4).

**Figure 2:**
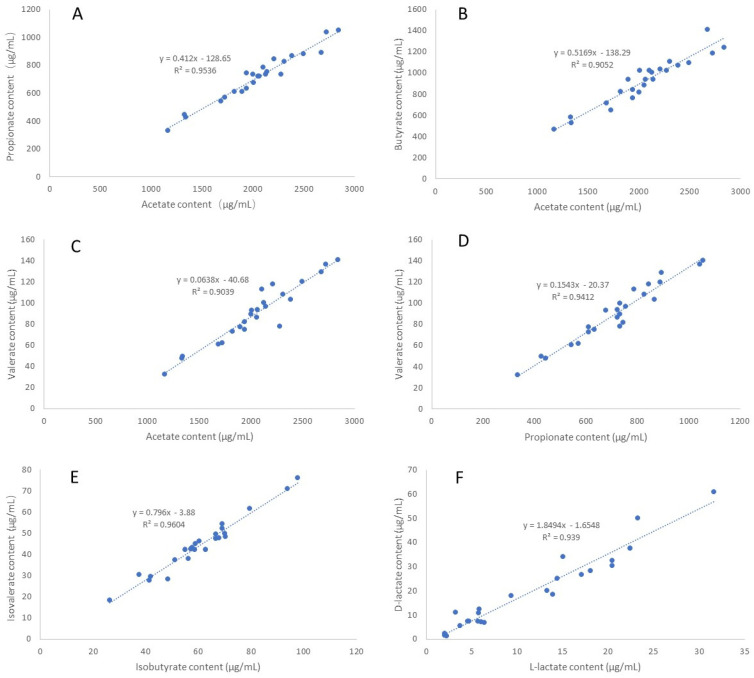
Correlations between D/L lactate and different species and isomers of SCFA in ruminal fluids (*n* = 24). (**A**) acetate vs. propionate; (**B**) acetate vs. butyrate; (**C**) acetate vs. valerate; (**D**) propionate vs. valerate; (**E**) isobutyrate vs. isovalerate vs. valerate; (**F**) L−lactate vs. D−lactate.

A pairwise correlation analysis was performed using the SCFA and D/L-lactate content data of the 24 individual samples. Strong correlations were observed between D- and L-lactate as well as between SCFA species and isomers, with the strongest correlated pairs being D-lactate/L-lactate, acetate/propionate, acetate/butyrate, acetate/valerate, propionate/valerate, and isobutyrate/isovalerate (Figure 2). By contrast, SCFA and D/L-lactate are not strongly correlated (r < 0.52, Table S2, Supporting Information).

## 3. Discussion

A robust yet simple method for simultaneous determination of all linear and branched SCFA and D/L-lactate enantiomers in ruminal fluid samples was established. Our method can replace the multiple methods previously reported for the quantification of SCFA and lactates in ruminal fluid.

Previously, SCFA were quantified separately to lactate, and two separate enzymatic assays were used to determine D- and L-lactate. To simplify the procedure, the simultaneous analysis of SCFA and D/L-lactate by LC-MS after a single derivatisation step was our first thought. However, whether the same derivatisation protocol previously optimised for SCFA could be applied to lactate was unknown, and what type of LC-MS system would be suitable for separating SCFA isomers and D/L-lactate isomers has not been established.

Our systematic investigation demonstrated that the 3-NPH derivatisation method for SCFA is equally applicable to lactate quantification. However, although being able to adequately resolve isobutyrate and butyrate, as well as the three isomers of valerate (2-methylbutyrate, isovalerate, and valerate) encountered in bovine milk and serum [14], RP columns cannot separate the chiral isomers, D-lactate and L-lactate Given that information on both D- and L-lactate is often required for understanding the microbial activity in the rumen, a RP-LC-MS analytical system was not suitable for a combined method.

Chiral-LC-MS has been used previously for determining D- and L-lactate in various biological samples. We have found that the chiral column also allows all the butyrate and valerate isomers to be baseline resolved within a total runtime of 20 min, enabling simultaneous determination of all SCFA and D/L-lactate in a single LC-MS run. In addition, the derivatised SCFA and D/L-lactate can be detected in both positive and negative ionisation modes with a similar sensitivity, providing flexibility in the MS setup options. 

Compared with non-derivatised SCFA [21], our method shows greater sensitivity and selectivity and affords better peak shape. Due to the high abundance of SCFA and D/L-lactate in ruminal fluid, an LOD of 0.01 µg/mL is more than adequate for such samples, so no attempt was made to further improve the MS response (e.g., using a narrower column and reducing the elution flowrate). Indeed, the ruminal samples had to be diluted prior to derivatisation due to the very high concentration of acetate and butyrate that falls firmly outside of the linear range of the MS detector. 

Our method showed excellent repeatability or precision for both intra-day and inter-day measurements, implying that both the derivatisation and the LC-MS steps are highly reproducible. Moreover, the close to 100% recovery of spiked analytes further demonstrate that no significant loss of analytes due to irreversible binding to the matrix nor ion suppression caused by the matrix, a phenomenon frequently encountered in electrospray ionization MS analysis. The synthetic SCFA analogue crotonate was used in the spike-recovery test to avoid the interference of highly abundant acetate, propionate, and butyrate in the ruminal fluid matrix. With a similar structure and property as those of SCFA, crotonate spiked in the matrix is expected to mimic the behaviour of SCFA while circumventing the complexity caused by endogenous SCFA. In addition, two dilutions of the matrix (20-fold and 40-fold) were tested to cover the possible scenarios in sample processing.

It is worth mentioning that the content of acetate and butyrate in the ruminal fluid is so high that even after a 40-fold dilution they may still be outside the linear range. In this case, the further dilution of the samples can be made after derivatisation, as no difference in the results was observed between the pre- and post-derivatisation dilution of samples. 

The application of the method to the quantification of SCFA and lactate in 24 individual animal ruminal samples allowed us to reveal the huge difference in abundance across different SCFA species as well as the large inter-cow variation in lactate content. Widespread correlations observed between different species of SCFA and between D- and L-lactate in ruminal fluid seem to suggest the close metabolic link between these molecules in the rumen.

## 4. Materials and Methods

### 4.1. Ruminal Fluid Samples

Samples of ruminal fluid were collected from 24 lactating, multiparous, Holstein Friesian cows with an average milk yield of 38.0 ± 3.65 kg milk/d (mean ± standard deviation), at 3.4 ± 1.65 parities, 42 ± 12.9 days in milk, and with a liveweight of 578 ± 59.1 kg. All cows were offered a common diet of 6.1 kg DM/d of a grain mix consisting of rolled barley grain (458 g/kg DM), solvent extracted canola meal (236 g/kg DM), rolled wheat grain (236 g/kg DM), molasses (15.0 g/kg DM), sodium bicarbonate (15.0 g/kg DM), limestone (13.0 g/kg DM), and minerals (27.0 g/kg DM), and grazed perennial ryegrass (*Lolium perenne* L.) pasture at an allowance of ~25 kg DM/d.

A single sample of ruminal fluid (~400 mL) was collected from each cow via esophageal tube at 4 h after the start of feeding. The 4 h delay from the start of feeding was chosen to coincide with the expected nadir in ruminal pH [22]. An oro-ruminal sampling probe, similar to that described by Geishauser [23], and a vacuum pump were used to collect samples [24]. Sub samples of 10 mL were transferred to 15 mL plastic vials without preservatives then stored frozen at −80 °C until analysis.

All procedures were conducted in accordance with the Australian Code of Practice for the Care and Use of Animals for Scientific Purposes [25]. Approval to conduct the experiment was obtained from the DEECA Agricultural Research and Extension Animal Ethics Committee (approval 2022—8 23 August 2022).

### 4.2. Chemicals

The chemicals and solvents used were of chromatographic/analytical grade. Standards of SCFA (acetate, propionate, isobutyrate, butyrate, 2-methylbutyrate, isovalerate, valerate, and crotonic acid), standards of D-lactate and L-lactate, SCFA derivatization reagents 3-nitrophenylhydrazine hydrochloride (3-NPH·HCl), 1-ethyl-3-(3-dimethylaminopropyl) carbodiimide (EDC), and pyridine were purchased from Sigma-Aldrich. Solvents (methanol) used for SCFA sample preparation and LC-MS mobile phase (0.1% formic acid in water and 0.1% formic acid in acetonitrile) were purchased from Fisher Scientific.

### 4.3. Derivatization

Lactate and SCFA derivatization method for ruminal fluid sample was adapted from that described previously for milk and serum samples [8]. Briefly, ruminal fluid samples were centrifuged for 15 min (13,000 *g*) at room temperature and the supernatant used for assay. All samples were diluted with 75% methanol and all derivatization reagents (pyridine, EDC, and 3-NPH) were dissolved in 75% methanol to the required concentrations. The derivatization reaction was carried out with 100 µL of sample, 50 µL of 6% pyridine, 50 µL of 50 mM EDC, and 50 µL of 50 mM 3-NPH at 30 °C for 60 min. After cooling to room temperature, the samples were diluted by adding 750 µL of milli-Q water before LC-MS analysis.

### 4.4. LC-MS Conditions

Derivatized SCFA and the lactate of ruminal fluid samples were separated by a Chiralpak IF-3 column (250 × 4.6 mm, 3 µm, Daicel Corporation, Osaka, Japan) on a Vanquish UHPLC system (Thermo Fisher Scientific, Waltham, MA, USA) with column compartment maintained at 30 °C and sample tray maintained at 15 °C. The mobile phase consisted of water containing 0.1% formic acid/acetonitrile containing 0.1% formic acid (50:50, *v*/*v*). An isocratic elution was adopted with a flow rate of 0.5 mL/min and an injection volume of 5 µL.

Derivatized lactate and SCFA were detected using an Orbitrap Elite mass spectrometer (Thermo Fisher Scientific, Waltham, MA, USA) with a heated electrospray ionisation (HESI) source. Capillary and source heater temperatures were 300 °C, and the sheath gas was at 40 units, auxiliary gas at 15 units, and sweep gas at 5 units. The mass spectrometer was operated in negative (−3.6 kV) ionization mode with a full scan (120 to 1800 *m/z*) at a resolution of 60,000. Lactate and SCFA in the samples were identified using Xcalibur (Thermo Fisher Scientific, Waltham, MA, USA) based on retention time and accurate mass matching.

### 4.5. Method Validation

The limit of detection (LOD), limit of quantification (LOQ) and linear range of the method were determined as previously described [14]. The method repeatability and reproducibility were estimated by intra-day and inter-day precision tests, respectively. The intra-day precision was assessed by analyzing four random ruminal fluid samples three times on the same day and calculating the relative standard deviation (RSD) for each analyte and each sample across the three analyses. The inter-day precision was evaluated by analyzing these same samples 10 days later and comparing the results of two time points.

The method reliability was also evaluated using the spike recovery test. To estimate the recovery of SCFA and D/L-lactate in the ruminal fluid matrix, a known amount crotonate (a SCFA analogue) and D-lactate standards (both dissolved in 75% methanol) was spiked into the ruminal fluid matrix before the derivatization reaction. The concentrations of crotonate and D-lactate in the spiked samples were then determined using the same protocol. Recovery rate (%) was calculated using the following formula:

Recovery (%) = total analyte found − analyte in the matrix/mass of analyte spiked × 100

The recovery rate was determined for two levels (0.625 and 2.5 of µg/mL) of crotonate and D-lactate standards spiked into two dilutions of ruminal fluid matrix (20-fold and 40-fold).

### 4.6. Method Applications

Twenty-four ruminal fluid samples from individual dairy cows were analyzed using our new method. The concentration range and the inter-cow variation for each SCFA; D/L-lactate was determined and the correlation across these acid species explored. All samples were diluted 40-fold before derivatization.

## 5. Conclusions

In conclusion, we validated a unified derivatisation and chiral-LC-MS method for the simultaneous quantification of SCFA and D/L-lactate in ruminal fluid. This method, without any sample pretreatment or purification step, is simple, robust, and suitable for processing ruminal samples for large-cohort experiments or repeated measures from animals.

## Figures and Tables

**Table 1:** Calculated mass, measured mass, limit of detection (LOD), limit of quantification (LOQ), linear range and regression coefficient (R^2^) of derivatised SCFA and D/L-lactate.

Name	Calculated Mass (*m/z*)	Measured Mass (*m/z*)	Mass Error (ppm)	Linear Range (µg/mL)	R^2^
Acetate	194.0566	194.0560	-3.09	0.033 to 20	1.0000
Propionate	208.0723	208.0717	-2.88	0.033 to 20	1.0000
Isobutyrate	222.0879	222.0872	-3.15	0.033 to 20	0.9999
Butyrate	222.0879	222.0872	-3.15	0.033 to 20	0.9999
2-methylbutyrate	236.1036	236.1029	-2.96	0.033 to 20	0.9999
Isovalerate	236.1036	236.1029	-2.96	0.033 to 20	0.9999
Valerate	236.1036	236.1029	-2.96	0.033 to 20	0.9999
Hexanoate	250.1192	280.1185	-2.80	0.033 to 20	1.0000
D-lactate	224.0672	224.0666	-2.68	0.033 to 20	0.9999
L-lactate	224.0672	224.0666	-2.68	0.033 to 20	0.9979

LOD: 0.01 µg/mL for all analytes; LOQ: 0.033 µg/mL for all analytes.

**Table 2:** Mean concentrations (µg/mL) and relative standard deviation (RSD%, *n* = 3) of short-chain fatty acids and D/L-lactate in four ruminal fluid samples.

Name	Sample 1		Sample 2		Sample 3		Sample 4	
	Mean	RSD	Mean	RSD	Mean	RSD	Mean	RSD
Acetate	2295.8	0.1	1388.0	1.1	1981.8	0.6	2078.1	1.1
Propionate	861.8	0.2	452.9	1.0	670.8	0.8	791.4	1.0
Isobutyrate	58.0	2.5	35.0	4.0	53.4	1.2	56.9	0.7
Butyrate	1054.9	0.3	535.9	1.4	760.9	0.8	842.0	0.3
2-methylbutyrate	46.6	1.0	27.8	0.7	71.9	1.3	60.0	1.0
Isovalerate	45.3	1.3	29.3	0.7	40.9	0.8	43.1	0.8
Valerate	124.3	1.4	52.4	0.7	80.3	0.8	99.9	1.4
Hexanoate	37.9	1.0	14.1	1.2	16.2	1.4	31.8	0.6
D-lactate	21.3	0.9	17.3	2.1	59.1	0.9	4.2	2.3
L-lactate	9.5	4.3	6.4	0.7	29.9	1.8	6.9	4.3

**Table 3:** Recovery (%, mean ± SD, *n* = 3) of spiked crotonate and D-lactate from ruminal fluid matrix at two spike levels (low spike: 0.625 µg/mL each and high spike: 2.5 µg/mL).

Analyte	40-Fold Diluted Matrix		20-Fold Diluted Matrix	
	Low Spike	High Spike	Low Spike	High Spike
Crotonate	96.7 ± 1.1	100.0 ± 2.7	93.4 ± 0.6	94.5 ± 0.8
D-lactate	100.8 ± 4.6	102.7 + 2.8	88.2 ± 4.4	92.9 ± 1.3

**Table 4:** Concentration range (in descending order) and variation of short-chain fatty acids and lactate in ruminal fluid of dairy cows (*n* = 24).

Name	Concentration (µg/mL)	RSD (%)
Acetate	1165.4 to 2842.5	20.6
Butyrate	469.5 to 1417.8	24.9
Propionate	333.3 to 1055.6	24.8
Valerate	32.3 to 140.6	31.4
Isobutyrate	26.3 to 97.7	26.5
Isovalerate	18.5 to 76.1	29.4
2-methylbutyrate	17.1 to 81.3	34.4
Hexanoate	10.4 to 44.9	37.9
L-lactate	2.0 to 31.6	73.0
D-lactate	1.2 to 60.9	81.8

## Data Availability

Data are contained within the article and supplementary materials.

## Supplementary Materials

The following supporting information can be downloaded at: https://www.mdpi.com/article/10.3390/molecules29061398/s1, Figure S1: Standard curves for short-chain fatty acids and D/L-lactate; Table S1: Inter-day measurement precision of the method; Table S2: Pairwise correlations between short-chain fatty acids and D/L-lactate in ruminal fluid samples.
